# The potency and synergy of plant‐made monoclonal antibodies against the BA.5 variant of SARS‐CoV‐2

**DOI:** 10.1111/pbi.13980

**Published:** 2023-01-20

**Authors:** Haiyan Sun, Collin Jugler, Katherine Nguyen, Herta Steinkellner, Qiang Chen

**Affiliations:** ^1^ The Biodesign Institute Arizona State University Tempe Arizona USA; ^2^ School of Life Sciences Arizona State University Tempe Arizona USA; ^3^ Department of Applied Genetics and Cell Biology University of Natural Resources and Life Sciences Vienna Austria

**Keywords:** SARS‐CoV‐2, COVID‐19, Omicron, Monoclonal antibody (mAb), plant‐made antibody, neutralization synergy

Severe acute respiratory syndrome coronavirus 2 (SARS‐CoV‐2) infections continue to persist around the globe, amid ongoing emergences of variants that are immune evasive. This is often attributed to mutations in the viral spike (S) protein that allow for reduced or abolished binding of neutralizing antibodies. Furthermore, most monoclonal antibodies (mAbs) that have been developed as therapies for SARS‐CoV‐2 infection have also lost utility against the Omicron variant and its subvariants (VanBlargan *et al*., 2022). ACE2‐based therapeutics have the potential to overcome this challenge, as variants that forgo binding to ACE2 will also lost infectivity (Daniell, 2022). As for mAbs, a cocktail called Evusheld retains neutralizing capacity against currently circulating variants of Omicron and is composed of two mAbs, named tixagevimab and cilgavimab, with non‐overlapping epitopes on the receptor binding domain (RBD) of the S protein (Jugler et al., 2022; Wang *et al*., 2022). Here, we developed new versions of tixagevimab and cilgavimab in *Nicotiana benthamiana* and show that the plant‐made counterparts of these mAbs efficiently neutralize multiple omicron subvariants of SARS‐CoV‐2 including the BA.5 and BA.4.6 possibly with enhanced effector function.

We genetically fused the variable regions of tixagevimab and cilgavimab onto a human IgG_1_ backbone, without the mutations that eliminate the Fc receptor (FcγR) binding of Evusheld mAbs, followed by transient expression of the mAbs in transgenic ΔXFT *N. benthamiana* plants (Appendix S1), where the glycosyltransferases for the synthesis of plant‐specific xylose and core fucose N‐glycans have been knocked down by a stable RNAi mechanism (Strasser *et al*., 2008). Peak expression of plant‐made cilgavimab (pCilgavimab) reached approximately 726 μg of mAb/g of fresh leaf weight (FLW) 6 days after agroinfiltration, while plant‐made tixagevimab (pTixagevimab) expression peaked 1 day earlier to approximately 403 μg of mAb/g FLW (Figure 1a). Glycan analysis of the conserved Fc glycosite showed that both mAbs contain highly homogeneous N‐linked glycans (Figure 1c), with >95% carrying human‐like biantennary GnGn or a hybrid MGn glycoforms. This is in stark contrast to the typical heterogeneous glycan populations found on mammalian‐made therapeutic proteins (Yang *et al*., 2018) and allows a more consistent therapeutic mAb product. Both mAbs specifically recognized the RBD of SARS‐CoV‐2 and were able to neutralize the authentic parental Omicron variant (B.1.1.529) in a foci‐forming assay, with an IC_50_ of 2.23 μg/mL and 19.68 μg/mL, respectively (Figure 1b).

**Figure 1:**
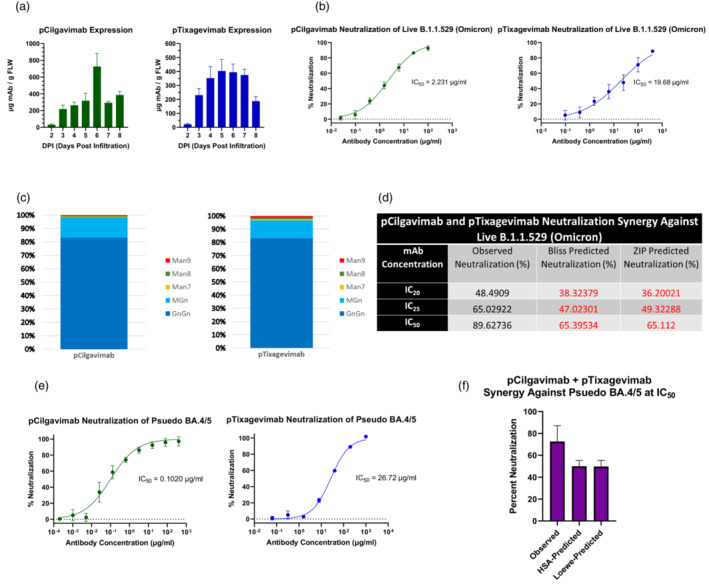
(a) Temporal expression of pCilgavimab and pTixagevimab in ΔXFT *N. benthamiana* plants. Data shown are in micrograms of mAb per gram of fresh leaf weight (FLW). DPI is days post‐agroinfiltration. (b) Neutralization analysis by foci‐forming assay for pCilgavimab and pTixagevimab against the live B.1.1.529 (Omicron) variant of SARS‐CoV‐2. The half maximal inhibitory concentration (IC_50_) value for each curve is indicated in the graph. Error bars are standard deviation and represent two independent experiments performed in triplicate. (c) Mass spectrometry analysis of pCilgavimab and pTixagevimab Fc glycosylation. Proportions of each glycan are represented as a percent of the total glycosylated Fc. GnGn, biantennary N‐actelyglucosamine; MGn, monoantennary N‐acetylglucosamine; Man9/8/7, mixed oligomannosidic structure. (d) Neutralizing synergy of the pCilgavimab and pTixagevimab cocktail against live B.1.1.529 (Omicron) variant. ZIP, zero interaction potency; IC_20_, IC_25_ and IC_50_: 20%, 25% and 50% inhibitory concentration, respectively. The predicated neutralization values were calculated from the neutralization data of each individual mAb in the cocktail and represent percent neutralization where there is no synergistic interaction between different mAbs. (e) Neutralization curves generated from a luciferase reporter assay for pCilgavimab and pTixagevimab against a BA.4/5 SARS‐Cov‐2 pseudovirus. IC_50_ value for each curve is indicated. (f) Percent neutralization of the combination of pCilgavimab and pTixagevimab at their respective IC_50_ concentrations against BA.4/5 SARS‐CoV‐2 pseudovirus. Both the observed and predicted percent neutralization from the HSA and Loewe synergy models are presented. Error bars in all graphs are standard deviation and represent at least two independent experiments performed in at least duplicates.

We also analysed the neutralization synergy of pCilgavimab and pTixagevimab as a dual mAb cocktail against live Omicron SARS‐CoV‐2. The mAbs were mixed, alongside individual mAb replicates at their respective IC_20_, IC_25_ and IC_50_ concentration and percent neutralization was determined. Empirically determined percent neutralization values for individual mAbs were then analysed in SynergyFinder.org using four models (Figure 1d) to calculate a predicted percent neutralization, which represents the percent neutralization of the cocktail assuming there is no synergistic interaction between the mAbs. Notably, pCilgavimab and pTixagevimab showed synergy at all three concentrations tested (Figure 1d) as indicated by consistent higher observed neutralization values than those predicted by all models. For example, synergy would be indicated by the Bliss and ZIP models at the IC_50_ concentration combination if observed percent neutralization was above 65.4% and 65.1%, respectively. Indeed, we observed 89.6% neutralization of the mAb IC_50_ combination, indicating synergetic neutralization was occurring against the authentic B.1.1.529 Omicron variant.

We further tested both plant‐made mAbs against the BA.4/5 Omicron subvariant using a pseudo virus‐based neutralization assay. Both pCilgavimab and pTixagevimab neutralized the BA.4/5 with IC_50_ values of 0.102 and 26.72 μg/mL, respectively (Figure 1e). This result is consistent with reports of mammalian made Evusheld mAbs, where cilgavimab has increased potency against BA.4/5 pseudovirus compared to previous Omicron variants, while tixagevimab has reduced potency against BA.4/5 (Wang *et al*., 2022). Furthermore, the neutralizing synergy observed between the two mAbs against the B.1.1.529 variant carried over to the BA.4/5 variant, where the combination of mAbs at their respective IC_50_ concentrations resulted in ~72.6% neutralization versus the non‐synergistic reference value of ~50% and ~49.8% predicted by the synergy models (Figure 1f). pCilgavimab and pTixagevimab also retained neutralization activity, albeit with reduced potency, against the latest dominant variant BA.4.6 (Table S1) which has been shown to be resistant to all other mAbs developed to date.

To our knowledge, this is the first report of the two mAbs that compose Evusheld being produced in plants and the very first synergy analysis of this cocktail against Omicron variants. Both mAbs reached high transient expression levels, reaching up to 403–726 mg/kg FLW. Furthermore, both mAbs neutralize multiple SARS‐CoV‐2 variants, with pCilgavimab having greater potency than pTixagevimab in neutralizing both the B.1.1.529 and BA.4/5 variants. As a combination, these mAbs show neutralizing synergy against the parental Omicron variant and remain efficacious against the BA.4/5 and BA.4.6 variants. Compared to the parent mAbs, pCilgavimab and pTixagevimab restored the FcγR binding and contained highly homogeneous, human‐like glycans. Given that therapeutic mAbs against SARS‐CoV‐2 require Fc effector functions for optimal protection and that GnGn glycoform increases binding to certain Fc receptors on immune cells (Winkler *et al*., 2021) but without introducing the risk of antibody‐dependent enhancement of infection (Dent *et al*., 2016), we speculate that these plant‐made mAbs have potentially increased therapeutic efficacy over their mammalian‐made counterparts. Our work sets the foundation for plants to serve as a particularly suitable platform for developing consistent, efficacious mAbs as SARS‐CoV‐2 therapeutics.

## Conflict of interest

The authors declare no conflicts of interest.

## Author contributions

QC conceptualized and directed the research. HYS and HS designed the experiments and analysed the data. HYS and KN performed the experiments. CJ drafted the manuscript with revisions by QC and HS.

## Supporting information


**Appendix S1** Expression and purification of cilgavimab and tixagevimab in *N. benthamiana* plants.
**Table S1** Half‐maximal inhibitory concentrations (IC_50_) of pTixagevimab and pCilgavimab against Omicron BA.4.6 pseudovirus.
