# Supplementary material for: The potency and synergy of plant‐made monoclonal antibodies against the BA.5 variant of SARS‐CoV‐2
Source: Plant Biotechnol J. 2023 Jan 20;21(3):463–5. doi: 10.1111/pbi.13980 (PMC9877843; doi:10.1111/pbi.13980)
Supplement: Supplementary file 1 — Appendix S1 Expression and purification of cilgavimab and tixagevimab in N. benthamiana plants. Table S1 Half‐maximal inhibitory concentrations (IC50) of pTixagevimab and pCilgavimab against Omicron BA.4.6 pseudovirus. [file PBI-21-463-s001.pdf]

Methods S1: Expression and purification of cilgavimab and tixagevimab in *N. benthamiana* plants.

Variable region sequences for light chain (LC) and heavy chain (HC) of cilgavimab and tixagevimab from literature (Dong et al., 2021) were synthesized by GENEWIZ and were genetically fused to a human kappa constant sequence of the LC and a human gamma IgG1 constant sequence of HC (Lai et al., 2010). LC and HC genes were then cloned into a bean yellow dwarf virus (BeYDV)-based geminiviral vector and transiently expressed in *N. benthamiana* plants by agroinfiltration as described previously (Chen and Lai, 2014).

7D11 mAb were extracted and purified according to published protocols (Jugler et al., 2020). Briefly, mAb-expressing *N. benthamiana* leaves 7 days post infiltration were harvested and homogenized in extraction buffer (1X phosphate-buffered saline, pH 5.2 with 10 mg/mL Na-L-ascorbate, 2mM phenylmethylsulfonyl fluoride, and 1mM ethylenediaminetetraacetic acid). The pH of the homogenate was adjusted to 5.2 and then centrifuged several times. The supernatant was filtered through a 0.2-micron vacuum filter. The clarified protein extract was subsequently subjected to Protein A (MabSelect, Cytiva) chromatography according to the manufacture's protocol prior to further analysis.

Chen, Q. and Lai, H. (2014) Gene delivery into plant cells for recombinant protein production. *Biomed Res Int* **2014**, 10.

Dong, J., Zost, S.J., Greaney, A.J., Starr, T.N., Dingens, A.S., Chen, E.C., Chen, R.E., Case, J.B., Sutton, R.E., Gilchuk, P., Rodriguez, J., Armstrong, E., Gainza, C., Nargi, R.S., Binshtein, E., Xie, X., Zhang, X., Shi, P.-Y., Logue, J., Weston, S., McGrath, M.E., Frieman, M.B., Brady, T., Tuffy, K.M., Bright, H., Loo, Y.-M., McTamney, P.M., Esser, M.T., Carnahan, R.H., Diamond, M.S., Bloom, J.D. and Crowe, J.E. (2021) Genetic and structural basis for SARS-CoV-2 variant neutralization by a two-antibody cocktail. *Nature Microbiology* **6**, 1233-1244.

Jugler, C., Joensuu, J. and Chen, Q. (2020) Hydrophobin-Protein A Fusion Protein Produced in Plants Efficiently Purified an Anti-West Nile Virus Monoclonal Antibody from Plant Extracts via Aqueous Two-Phase Separation. *International Journal of Molecular Sciences* **21**, 2140.

Lai, H., Engle, M., Fuchs, A., Keller, T., Johnson, S., Gorlatov, S., Diamond, M.S. and Chen, Q. (2010) Monoclonal antibody produced in plants efficiently treats West Nile virus infection in mice. *Proc. Natl. Acad. Sci. U S A* **107**, 2419-2424.

Variant	pTixagevimab(IC_{50})	pCilgavimab (IC_{50})
**Omicron BA.4.6**	90.65 µg/ml	568.30 µg/ml

Table S1. Half-maximal inhibitory concentrations (IC_{50}) of pTixagevimab and pCilgavimab against Omicron BA.4.6 pseudovirus.
